# Quality of Antimalarial Drugs and Antibiotics in Papua New Guinea: A Survey of the Health Facility Supply Chain

**DOI:** 10.1371/journal.pone.0096810

**Published:** 2014-05-14

**Authors:** Manuel W. Hetzel, Madhu Page-Sharp, Nancy Bala, Justin Pulford, Inoni Betuela, Timothy M. E. Davis, Evelyn K. Lavu

**Affiliations:** 1 Papua New Guinea Institute of Medical Research, Goroka, EHP, Papua New Guinea; 2 Swiss Tropical and Public Health Institute, Basel, Switzerland; 3 University of Basel, Basel, Switzerland; 4 Curtin University, School of Pharmacy, Perth, WA, Australia; 5 Central Public Health Laboratory, Boroko, NCD, Papua New Guinea; 6 The University of Queensland, School of Population Health, Herston, QLD, Australia; 7 Papua New Guinea Institute of Medical Research, Madang, MDG, Papua New Guinea; 8 University of Western Australia, School of Medicine and Pharmacology, Fremantle, WA, Australia; Tulane University School of Public Health and Tropical Medicine, United States of America

## Abstract

**Background:**

Poor-quality life-saving medicines are a major public health threat, particularly in settings with a weak regulatory environment. Insufficient amounts of active pharmaceutical ingredients (API) endanger patient safety and may contribute to the development of drug resistance. In the case of malaria, concerns relate to implications for the efficacy of artemisinin-based combination therapies (ACT). In Papua New Guinea (PNG), *Plasmodium falciparum* and *P. vivax* are both endemic and health facilities are the main source of treatment. ACT has been introduced as first-line treatment but other drugs, such as primaquine for the treatment of *P. vivax* hypnozoites, are widely available. This study investigated the quality of antimalarial drugs and selected antibiotics at all levels of the health facility supply chain in PNG.

**Methods and Findings:**

Medicines were obtained from randomly sampled health facilities and selected warehouses and hospitals across PNG and analysed for API content using validated high performance liquid chromatography (HPLC). Of 360 tablet/capsule samples from 60 providers, 9.7% (95% CI 6.9, 13.3) contained less, and 0.6% more, API than pharmacopoeial reference ranges, including 29/37 (78.4%) primaquine, 3/70 (4.3%) amodiaquine, and one sample each of quinine, artemether, sulphadoxine-pyrimethamine and amoxicillin. According to the package label, 86.5% of poor-quality samples originated from India. Poor-quality medicines were found in 48.3% of providers at all levels of the supply chain. Drug quality was unrelated to storage conditions.

**Conclusions:**

This study documents the presence of poor-quality medicines, particularly primaquine, throughout PNG. Primaquine is the only available transmission-blocking antimalarial, likely to become important to prevent the spread of artemisinin-resistant *P. falciparum* and eliminating *P. vivax* hypnozoites. The availability of poor-quality medicines reflects the lack of adequate quality control and regulatory mechanisms. Measures to stop the availability of poor-quality medicines should include limiting procurement to WHO prequalified products and implementing routine quality testing.

## Introduction

Prompt and effective treatment of clinical episodes of malaria is one of the central pillars of malaria control programs [1]. It is critical for preventing progression to severe disease or death and reducing the reservoir of *Plasmodium* parasites in the population. The World Health Organization’s (WHO) “T3: Test. Treat. Track.” initiative emphasises that every confirmed case of malaria should be treated with an antimalarial medicine that is quality-assured and efficacious in the specific local setting [2].

The quality of antimalarial medicines has come under scrutiny after an increasing number of reports identified poor-quality products in malaria-endemic countries in Africa, Asia and Latin America [3]–[6]. A recently published review found that 35% of antimalarial drug samples from seven countries in southeast Asia and 21 countries in sub-Saharan Africa had failed chemical content analysis [3]. A number of studies found products sampled from unlicensed providers to be of poor quality more frequently than products from licensed providers (pharmacies, health facilities) [7]. While antimalarial drug quality has been studied more extensively, the problem extends to other anti-infective drugs, particularly antibiotics and anti-retrovirals, and beyond [7]–[9]. Estimates of the prevalence of poor-quality medicines vary widely as a result of a lack of surveillance and testing capacity, and disagreements between stakeholders on definitions and appropriate action [10], [11]. Resource-poor countries, in which regulatory and law enforcement systems are insufficient for detecting poor-quality products and preventing them from entering or remaining on the local medicines market, are likely to be most affected [12], [13]. The discovery of 1.4 million packets of fake Coartem (artemether-lumefantrine) hidden in loudspeaker boxes in a shipping container in Luanda (Angola) in 2012 may be an indication of the magnitude of the problem and of the criminal energy driving it [14].

Poor-quality life-saving medicines endanger patient safety and are recognised as a major public health threat [11], [13], [15], [16]. Poor-quality products can result from sub-standard manufacturing practices, or they may degrade due to inadequate transportation and storage conditions. The end result is products with less than the required amount of active pharmaceutical ingredient (API) [17]. Falsified medicines, on the other hand, are due to deliberate fraudulent activity and often contain only traces of or no relevant active ingredient at all. The term ‘counterfeit medicines’ is in this context generally used when referring to intellectual property rights infringements [17], [18].

The exposure of malaria parasites to sub-lethal amounts of antimalarial medicines may result in treatment failure and ultimately death of a patient but may also contribute to the development of drug resistance [19]. Particular concerns relate to the emergence of falsified artemisinin derivatives and potential implications for the efficacy of artemisinin-based combination therapies (ACT) [3]. In Papua New Guinea (PNG), where *Plasmodium falciparum* and *P. vivax* are both endemic [20], the National Department of Health has recently introduced ACT as first-line treatment [21]. However, other antimalarial medicines, such as primaquine for the treatment of *P. vivax* hypnozoites, are widely available [22].

Medicines can be obtained from a variety of sources but the over 4,000 formal health facilities (government, church and privately run hospitals, health centres and aid posts) represent the widest network of providers [23]. Two studies conducted primarily in rural settings of PNG reported that health facilities were the most common source of antimalarial treatment [24]–[26]. A national household survey carried out in 2008/09 found that 45% of recently febrile household members had attended a health facility and 74% of them had received an antimalarial medicine. Of the 55% not attending a health facility only 9% reported taking an antimalarial [25]. The situation of other anti-infective drugs, particularly common antibiotics, has not been thoroughly investigated. Malaria treatment can also be obtained from private licensed pharmacies, of which there were 76 in 2011, according to the PNG Pharmacy Board [27]. In certain places, street vendors, markets and retail stores may represent additional sources of medicines, but to the authors’ knowledge, the local market structure for medicines has never been thoroughly investigated. While dispensing of pharmaceuticals is limited to licensed providers, there are numerous gaps in the regulatory framework and its enforcement [27].

The supply of medicines to government and church-run health facilities follows a push-system centralised at the National Department of Health. Distribution to facilities relies on a network of regional warehouses (area medical stores, AMS) and provincial transit stores [27]. Delivery from provincial stores to individual facilities is the responsibility of provincial health offices/authorities. Individual disease control programs may circumvent the routine delivery procedures and church-run facilities may also obtain medicines through their own supply channels.

In the absence of routine quality control procedures [27], little is known about the quality of medicines dispensed in PNG. Only recently has the Global Fund Round 8 malaria grant [28] provided funding for establishing a Minilab (Global Pharma Health Fund e.V., Germany) testing facility at the Central Public Health Laboratory (CPHL). In 2011, a first small-scale study found substandard and counterfeit anti-infective medicines in private pharmacies in Port Moresby [29]. While these results are alarming, the evidence base is very limited and the data not representative for the country as a whole. The quality of medicines at the level of providers in rural areas may differ substantially from the quality found in the capital [30]. Similarly, medicines found on offer at different types of providers may be of different quality as the sources and storage conditions for drugs may vary.

This study aimed to investigate the quality of antimalarial medicines at all levels of the health facility drug supply chain in PNG in order to provide an understanding of the exposure of malaria patients to poor-quality products. At the time of the study, the new treatment protocol introducing artemether-lumefantrine (AL) as first-line treatment for uncomplicated *P. falciparum* malaria, and AL plus primaquine as first-line treatment for uncomplicated *P. vivax* malaria was in the early stages of implementation and AL had not yet been distributed to all health facilities. The study was extended to sampling two antibiotics as examples of anti-infective medicines frequently used for the treatment of non-malarial fevers. Where possible, this report follows the Medicine Quality Assessment Reporting Guidelines (MEDQUARG) [17].

## Methods

### Ethical Statement

The study protocol was reviewed and granted ethical clearance by the PNG Institute of Medical Research Institutional Review Board (IMR IRB No. 1115) and the PNG Medical Research Advisory Committee (MRAC No. 11.25). Verbal approval to collect medicine samples was obtained from the officers-in-charge of the respective providers.

### Product Sampling

Samples of medicines were collected in 2011 during a national cross-sectional health facility survey which included randomly sampled health centres, health sub-centres (here collectively referred to as health centres) and aid posts. Two health centres and up to four aid posts were randomly sampled from each of 20 provinces of PNG, using a simple random sampling procedure. The sampling frame was a list of all 689 operational public-sector health centres as provided by the National Department of Health. Aid posts were randomly selected from a list of all operational aid posts under the supervision of the health centre at the time of survey. The health facility survey was the second in a series carried out for the evaluation of the National Malaria Control Program and the survey methodology is described in more detail elsewhere [31].

Trained field interviewers from the Papua New Guinea Institute of Medical Research (PNG IMR) collected samples of a minimum of 30 tablets or capsules of medicines available in the facility, including separate samples of the same drug if the brand/manufacturer or dosage strength differed. The range of products included oral tablet/capsule formulations of all antimalarial medicines used in the previous and current malaria treatment guidelines [21], [32], [33] as well as amoxicillin and doxycycline (which is also used in treating malaria [34]). Samples were collected after full disclosure of the study objectives to the officer-in-charge of the respective facility. When required, field interviewers provided replacement medicines obtained from the AMS in the town of Lae. In addition to the health centres and aid posts, medicines were collected from a convenience sample of provincial hospitals and AMS following the same collection approach as described above.

Medicines were collected in their original blisters or jars, or, in case of large containers (e.g. containing 1,000 tablets), sampled into a plastic zip-lock bag. Each sample was labelled with a unique identifier. Details of each drug sample, such as API, dosage strength, manufacturer’s details, etc. were copied from the package into a data collection form. For each provider, basic information about their drug storage facility was recorded. Drug samples were stored dark in an air-conditioned room at the CPHL before being sent to Australia for chemical content analysis.

### Product Evaluation

Medicine samples were analysed for API content at Curtin University School of Pharmacy in Perth, Australia, using a high performance liquid chromatography (HPLC; Hewlett Packard model 1100) system comprised of a gradient pump, autosampler and a variable wavelength UV detector (Agilent Technology, Waldbronn, Germany). A five point calibration curve was generated for each drug. Each calibration curve demonstrated to be linear showing a regression coefficient r^2^≥0.999. Chromatogram analysis was performed using Chemstation Software (Version 9, Agilent Technology). Published HPLC methods were modified as required for each drug analysis (Table 1). Chemistry analyses were performed blinded to packaging of the medicines.

**Table 1:** HPLC method applied for each substance.

Drug [Reference]	Column	Mobile phase	Flow rate(ml/min)	λ_max_(nm)	Col T(°C)	Inj vol(µl)	Rt(min)
Chloroquine [56]	Gemini C6-phenyl 110A (150×4.6 mm 5 µm)	0.05M KH_2_PO_4_ pH 2.5+13% acetonitrile	1.2	343	30	20	3.8
Amodiaquine [56]	Gemini C6-phenyl 110A (150×4.6 mm 5 µm)	0.05M KH_2_PO_4_ pH 2.5+13% acetonitrile	1.2	343	30	20	5
Sulphadoxin/Pyrimethamine [57]	Gemini C6-phenyl 110A (150×4.6 mm 5 µm)	0.1% H_3_PO_4_+23% acetonitrile	1	227	30	20	2.1/6.9
Primaquine phosphate [58]	Apollo C_18_ (150×4.6 mm, 5 µm)	0.05M H_3_PO_4_ pH 3+28% acetonitrile	1	254	30	20	2.5
Quinine sulphate [59]	Gemini C6-phenyl 110A (150×4.6 mm 5 µm)	0.05M H_3_PO_4_ pH 3+30% acetonitrile	1	340	30	20	2.2
Amoxicillin tablets/capsules [60]	Apollo C_18_ (150×4.6 mm, 5 µm)	0.05M H_3_PO_4_ pH 3+8%v/v acetonitrile	1.2	272	25	20	3.5
Doxycycline hyclate [61]	Gemini C6-phenyl 110A (150×4.6 mm 5 µm)	Water +0.1% TFA+acetonitrile +0.1%TFA (62∶38)	1	360	25	20	3
Artesunate [62]	Apollo C_18_ (150×4.6 mm, 5 µm)	0.05M H_3_PO_4_ pH 3+70% acetonitrile	1	220	25	50	2.8
Artemether [63]	Apollo C_18_ (150×4.6 mm, 5 µm)	Water +85% acetonitrile	1.5	220	25	50	3.5
Lumefantrine [64]	Gemini C6-phenyl 110A (150×4.6 mm 5 µm)	0.05M acetate buffer pH 2.5+80% acetonitrile	1	335	25	20	3

#### Sample preparation and analysis techniques

Standards and samples of each drug were prepared using the diluents listed in Table 2. The reference drugs were obtained from Sigma-Aldrich, Castle Hill, Australia (amoxicillin, artesunate, chloroquine diphosphate, doxycycline, primaquine diphosphate, sulphadoxine & pyrimethamine), Sigma Chemicals, Perth, Western Australia (quinine hydrochloride), Sigma Chemicals, St Louis, MO, USA (amodiaquine dihydrochloride dihydrate), AAPIN Chemicals Ltd Abingdon, UK (artemether) and Shaani Sciphar, Biotechnology, Chime Co. Ltd., China (lumefantrine). Standard concentrations were prepared at 1 mg/mL. All stock solutions were stored at 4°C. Chloroquine and amodiaquine were analysed for individual tablet content. Six individual tablets from each batch were weighed, dissolved and analysed individually. For the other drugs, 3–20 tablets from each batch were weighed to obtain the average tablet weight (Table 2). The tablets were then crushed to fine powder. Each sample was prepared by dissolving the amount of powder equivalent to the average weight of one tablet. The solutions were sonicated for 10 min × 2 and then equilibrated to room temperature for 30 minutes. A known volume of the clear solution of the first dilution was assayed or further dilutions were done if required to remain within the assay limit (Table 2).

**Table 2:** Standard and sample preparations for HPLC assay.

Drug	Tablet strength (mg)	Tablets per batch	Diluent	1^st^ dilution (ml)	2^nd^ dilution factor
Amodiaquine	100	6	Water	100	2
Amoxicillin capsules	500	20	0.1% H_3_PO_4_	100	10
Amoxicillin tablets	250	20	0.1% H_3_PO_4_	100	5
Artemether	50	6–20	Acetonitrile	100	–
Artemether-lumefantrine	20/120	3–20	Acetonitrile +2% acetic acid	100	2
Artesunate	50	6–20	Acetonitrile	100	–
Chloroquine	150	6	Water	100	3
Doxycycline hyclate	100	20	0.01M HCL	100	5
Primaquine phosphate	7.5/13.2/26.3	20	Water +0.1% formic acid	100	–
Quinine sulphate	300	20	Methanol	100	5
Sulphadoxin-pyrimethamine	500/25	20	Acetonitrile	200	5

#### Chemical content analysis of primaquine tablets by LC-MS

Primaquine tablet samples were also tested by liquid chromatography-mass spectrometry (LC-MS) for quality control purposes. The LC-MS and chromatographic conditions were consistent with previously published methods [35]. The single-quad LC-MS system (model 2020, Shimadzu, Kyoto, Japan) was used for the analysis. Separation was performed in isocratic mode using a Luna C18 (100 mm × 4.6 mm i.d., 3 µm) column, (Phenomenex, Lane Cove, Australia). Mobile phase consisting of methanol:water (80∶20 v/v) with 0.1% formic acid was pumped with a flow rate of 0.25 ml/min. Primaquine and 8-aminoquinoline was scanned first in ESI positive mode to identify the abundance of ions. Quantitation was performed by selected ion monitoring in ESI positive mode using protonated parent molecule [M = H]+; m/z 260 for primaquine and protonated parent molecule [M = H]+; m/z 145 for 8-aminoquinoline. A five point calibration from 1–7 µg/ml was constructed in 0.1% formic acid and each standard and batch sample was spiked with 0.5 µg of 8 aminoquinoline (IS). The standard curve was linear with r^2^ = 0.99. Each batch of tablets was injected in random order along with the quality control samples. Data was processed using LAB Solution (Version 5, Shimadzu, Japan).

### Data Analysis

Questionnaire data were double-entered into a DMSys database (version 5.1, Sigma Soft International) at PNG Institute of Medical Research and linked to HPLC assay results in Stata (version 12.1, StataCorp) which was also used for data analysis.

A sample was considered to have failed chemical content analysis if the measured percentage of API was below or above the acceptable range defined in the British, International, or US Pharmacopoeia (reference values provided in Table 3). Differences in primaquine content detected by LC-MS and HPLC were ≤8.3% but minor differences in samples near the lower reference threshold (93%) resulted in fewer samples failing the LC-MS assay. In the results, the LC-MS data are presented but comparative data of both methods are provided as Table S1. Content of active ingredient of a samples (µg/ml) was calculated from standard curve × dilution (25*100) × (average tablet weight/assay weight)/1000 = total drug in each sample (mg).

**Table 3:** Acceptable ranges of active pharmaceutical ingredients and respective references.

Active ingredient	Acceptable range (% active ingredient)*	Reference
Amodiaquine	95.0–105.0	British Pharmacopoeia (BP)
Amoxicillin capsule 500 mg	92.5–110.0	British Pharmacopoeia (BP)
Amoxicillin tablet 250 mg	90.0–110.0	British Pharmacopoeia (BP)
Artemether	90.0–110.0	British Pharmacopoeia (BP)
Artesunate	90.0–110.0	International Pharmacopoeia (IP)
Chloroquine	92.5–107.5	British Pharmacopoeia (BP)
Doxycycline hyclate 100 mg	95.0–105.0	British Pharmacopoeia (BP)
Lumefantrine	90.0–110.0	British Pharmacopoeia (BP)
Primaquine	93.0–107.0	United States Pharmacopoeia (USP)
Pyrimethamine	90.0–110.0	British Pharmacopoeia (BP)
Quinine sulphate	95.0–105.0	British Pharmacopoeia (BP)
Sulphadoxine	90.0–110.0	British Pharmacopoeia (BP)

*Relative to labelled amount.

Univariate analysis was performed to describe basic characteristics of the samples. The outcomes of the chemical content analyses (pass/fail) were compared across regions, type of provider, and other co-variates. Bivariate analyses included Fisher’s exact and chi-square tests. Multilevel models with random effect were fitted to assess associations of individual sample content analysis outcome and country of origin, provider type and storage conditions taking into consideration possible clustering at a provider-level. Multivariable logistic regression was used to assess the association of storage conditions and facility-level availability of poor-quality drugs.

## Results

### Sources and Types of Samples

The survey covered 60 providers, including 12 hospitals, 17 health centres, 22 aid posts, 4 urban clinics (collectively referred to as health facilities) and 5 AMS. Providers were located in 18/20 provinces. Delays in the ethical approval process prevented the collection of samples in the first provinces covered by the health facility survey (New Ireland, West New Britain).

A total of 392 medicine samples in the form of tablets or capsules were collected, 385 of which were available in sufficient quantity to be analysed for API content by HPLC/LC-MS. Of these, 6 (one amodiaquine, one chloroquine, four primaquine) had expired before 2011, the year of collection, 16 (one SP, one chloroquine, four quinine, four primaquine, two artesunate, four doxycycline) had expired between collection and the time of performing the chemical content analysis in 2012, and for two samples (one primaquine, one artemether), no expiry date was available. These samples were analysed separately and results presented as supplementary information (Tables S1 and S2). A single sample of quinidine was excluded from analysis.

### Content of Active Pharmaceutical Ingredient

Of 360 unexpired samples, 37 (10.3%; 95% confidence interval [CI] 7.3, 13.9) failed the chemical content analysis. Two failed samples of quinine (0.6% of all samples, 95% CI 0.1, 2.2) contained more than the acceptable amount of API while 35 samples (9.7%; 95% CI 6.9, 13.3) contained less API than acceptable (as defined in Table 3). In all of the tested samples some active ingredient was detected. Failed samples included 29 (78.4%) primaquine, 3 (4.3%) amodiaquine, 2 (6.3%) quinine, 1 (4.5%) artemether, 1 (1.8%) SP and 1 (4.3%) amoxicillin capsule sample (Table 4). The failed primaquine samples contained on average 70.7% (standard deviation [SD] 10.9) of API, the failed amodiaquine samples 45.2% (SD 5.4). The two failed quinine samples contained 106.5% and 105.2% API. Details of all failed samples are presented as Table S2.

**Table 4:** Chemical content analysis results by active pharmaceutical ingredient.

Drug	No. of samples tested	% active ingredient			Failed		
		mean	min	max	Below range	Above range	Total
Amodiaquine tablets	70	97.1	41.6	103.0	3	0	3 (4.3%)
Amoxicillin capsules	23	96.2	91.8	102.2	1	0	1 (4.3%)
Amoxicillin tablets	24	95.1	91.5	101.3	0	0	0
Artemether tablets	22	96.7	88.6	109.4	1	0	1 (4.5%)
AL tablets: Artemether	9	97.6	94.9	103.6	0	0	0
AL tablets: Lumefantrine	9	102.0	91.4	106.8	0	0	0
Chloroquine tablets	76	98.7	94.9	104.3	0	0	0
Doxycycline tablets	12	99.1	95.3	102.1	0	0	0
Primaquine tablets	37	75.9	60.0	98.7	29	0	29 (78.4%)
Quinine tablets	32	100.1	95.3	106.5	0	2	2 (6.3%)
SP tablets: Sulphadoxine	55	95.6	90.9	103.9	0	0	0
SP tablets: Pyrimethamine	55	97.2	89.4	107.2	1	0	1 (1.8%)
**Total**	**360**				**35**	**2**	**37 (10.1%)**

AL = Artemether-lumefantrine, SP = Sulphadoxine-pyrimethamine.

### Labelled Origin of Samples

The majority of the medicines was collected in packages that were un-opened or sealed (where applicable) at the time of collection (83.1% of collected samples, 81.1% of failed samples). According to the package labels, most of the 360 analysed samples were manufactured in China (57.2%) and India (40.8%) (Table 5). The remaining samples originated from Indonesia (5) and Switzerland (1). The origin of one sample could not be ascertained on the basis of the manufacturer name recorded by the field data collector. Most of the 37 samples that failed the chemical content analysis had been manufactured in India (86.5%; 95% CI 71.2, 95.5) according to the package label (Table 5). The remaining failed samples originated from China (4) and Indonesia (1). In total, 21.8% (32/147) of the samples from India and 1.9% (4/206) of the samples from China failed the chemical content analysis. The difference between samples from India and China was partly explained by the large proportion of primaquine samples that were labelled as originating from India (89.2%). After adjusting for primaquine, the difference between samples from India and China remained statistically significance but with a wide confidence interval (adjusted odds ratio [AOR] = 4.4, 95% CI 1.0, 19.6, p = 0.048).

**Table 5:** Origin of analysed and failed samples according to package labels.

Country	Analysed samples	Failed samples
	N (%)	N (%)
China	206 (57.2)	4 (10.8)
India	147 (40.8)	32 (86.5)
Indonesia	5 (1.4)	1 (2.7)
Switzerland	1 (0.3)	
Unclear	1 (0.3)	
**Total**	**360**	**37**

The 360 samples originated from a total of thirteen manufacturers, according to the package labels. Only two manufacturers’ products (artemether-lumefantrine from Ipca, India and Novartis, Switzerland) were WHO prequalified [36] and both passed the content analysis. The 37 failed samples were manufactured by nine different companies. In the case of three manufacturers, all collected samples failed the chemical content analysis (Table 6). The degree to which the measured API content deviated from the acceptable range differed between the manufacturers. Products with particularly low levels of API originated from three companies (Bharat Parenterals, Dev Life Corporation and Trends Pharma) (Table S2).

**Table 6:** Manufacturers of analysed and failed tablet/capsule samples according to package labels.

	Number of samples failed/Number collected (% failed)									
Manufacturer according to package label	AQ	ART	AL	CQ	PQ	QU	SP	AMX	DOX	Total
Alken Laboratories, country unclear								0/1		0/1
Ally Pharma Options Pvt. Ltd., India						1/17 (5.9)	0/4			1/21 (4.8)
BDH Industries Ltd., India					6/10 (60.0)	0/10				6/20 (30.0)
Bharat Parenterals Ltd., India					6/6 (100)					6/6 (100)
Dev Life Corporation, India					16/16 (100)					16/16 (100)
Ipca Laboratories, India			0/8*	0/31						0/39
Kimia Farma, Indonesia						1/5 (20.0)				1/5 (20.0)
Kunming Pharmaceutical Corp, China		1/22 (4.5)								1/22 (4.5)
Medopharm, India				0/41						0/41
North China Pharmaceutical Group Corp., China	0/43			0/4			1/50 (2.0)	1/25 (4.0)	0/12	2/134 (1.5)
Novartis, Switzerland			0/1*							0/1
Shijiazhuang Pharma Group, China	0/24				1/4 (25.0)		0/1	0/21		1/50 (2.0)
Trends Pharma Pvt. Ltd., India	3/3 (100)									3/3
**Total**	**70**	**22**	**9**	**76**	**37**	**32**	**55**	**47**	**12**	**360**

AQ = amodiaquine, ART = artemether, AL = artemether-lumefantrine, CQ = chloroquine, PQ = primaquine, QU = quinine, SP = sulphadoxine-pyrimethamine, AMX = amoxicillin, DOX = doxycycline. Total column includes all samples from a particular manufacturer. *WHO prequalified products.

The authors contacted the manufacturers of unexpired failed samples in order to verify the authenticity of the package and with it the origin of the product. Manufacturers producing only one or two failed samples were not contacted. Representatives of four manufacturers (BDH Industries, Bharat Parenterals, Dev Life Corporation and Trends Pharma) confirmed the authenticity of the packages. Sample photographs of packages are provided as supplementary information (Figure S1).

### Poor-quality Products in the Health Facility Supply Chain

Medicines that failed content analysis were found at all levels of the supply chain, with the largest number collected from hospitals and health centres (Table 7). The proportion of failed samples was highest in area medical stores (5/22, 22.7%) and hospitals (14/88, 15.9%), and lower in health centres (10/120, 8.3%), urban clinics (3/30, 10.0%) and aid posts (5/100, 5.0%). Differences between providers were largely explained by the number of primaquine samples collected and were not statistically significant in a multilevel analysis after adjusting for primaquine (p>0.1 for all provider types).

**Table 7:** Origin of analysed and failed samples within the supply chain.

	Analysed samples	Failed samples
Facility type	N (%)	N (%)
Area medical store	22 (6.1)	5 (13.5)
Hospital	88 (24.4)	14 (37.8)
Health centre	120 (33.3)	10 (27.0)
Aid post	100 (27.8)	5 (13.5)
Urban clinic	30 (8.3)	3 (8.1)
**Total**	**360**	**37**

Failed medicines were found in 16/18 surveyed provinces (except Western Province and Bougainville) and in 48.3% of the 60 providers sampled for this study, including all five area medical stores, 8/12 (66.7%; 95% CI 34.9, 90.1) hospitals, 8/17 (47.1%; 95% CI 23.0, 72.2) health centres, 5/22 (22.7%; 95% CI 7.8, 45.4) aid posts and 3/4 (75%; 95% CI 19.4, 99.4) urban clinics. The difference between types of providers was not statistically significant after correcting for primaquine in a multivariate logistic regression (all p>0.9). The facility-level analysis confirmed that the presence of poor-quality medicines was not correlated with the presence of a separate storage room (43/60; AOR = 0.94, 95%CI 0.18, 4.96, p = 0.95) or an air conditioning system for the storage room (17/60; AOR = 2.99, 95% CI 0.50, 17.69, p = 0.23) but explained largely by the presence of primaquine.

## Discussion

This is the first study documenting the presence of poor-quality medicines in the health facility supply chain throughout PNG. About half (48.3%) of all surveyed providers across the country were found to stock medicines that failed chemical analysis of the amount of API. This survey focused on antimalarial medicines and two antibiotics as examples of other anti-infective drugs. In conjunction with two earlier small-scale studies that detected substandard, falsified and counterfeit amodiaquine, amoxicillin, artemether and artesunate in samples collected from private pharmacies in Port Moresby [29], [37], this study confirms the presence of poor-quality medicines in both the public and private sectors in PNG.

While the majority of all medicine samples collected for this study was of good quality, most primaquine tablet samples (78.4%) failed the chemical content analysis. Most of the failed primaquine samples contained less than 70% API (i.e. <5.25 mg/tablet instead of 7.5 mg) and three amodiaquine samples contained between 41.6 and 51.3% API (i.e. 41.6–51.3 mg/tablet instead of 100 mg). At these levels, the drugs are unlikely to have a full therapeutic effect, but may contribute to the development of resistance of the parasites. On the other end of the spectrum, two samples of quinine containing over 105% API may exacerbate adverse effects in patients due to the drug’s narrow therapeutic index [38].

Primaquine plays a central role in the control of *P. vivax* malaria and, more generally, in current efforts focussed on malaria elimination [39]. Primaquine is the only drug currently available for the elimination of *P. vivax* and *P. ovale* hypnozoites, for which a 14-day regimen is currently recommended [1], [40]. Primaquine is also active against gametocytes of *P. falciparum,* including those persisting after treatment with ACT [41], for which purpose a single dose is considered sufficient [1], [42]. While there remains certain controversy around the impact of primaquine on malaria transmission [43], the drug is considered a potentially important tool against artemisinin-resistant *P. falciparum* malaria [42], [44]. Poor-quality primaquine, as identified in abundance in this study, may compromise the global strategy to contain the spread of artemisinin-resistant *P. falciparum* and efforts of eliminating *P. vivax*. At the same time, under-dosing of primaquine may provide false reassurance as to the incidence and severity of major drug-related adverse effects such as haemolytic anaemia in G6PD deficient patients, and methaemoglobinaemia [45], [46]. The evidence of the wide-spread availability of poor-quality primaquine found in this study alongside results from investigations in other locations [6], [47], [48] is therefore a concern for all countries in which primaquine should have a major role in malaria control, including settings in which the drug is already frequently used [48], [49].

Drug quality needs to be considered alongside aspects of safety in the discussion about the application of primaquine for reducing *P. falciparum* malaria transmission and radical cure of *P. vivax*. Broader confirmatory investigation into the quality of primaquine is an urgent priority as part of the assessment of measures of quality control and quality assurance in malaria control and research programs that use primaquine.

The results presented in this study are limited to chemical content analysis by HPLC and LC-MS and did not comprise dissolution testing, which would have provided complementary information related to the drugs’ bioavailability, particularly for poorly aqueous-soluble drugs such as sulphadoxine-pyrimethamine [50]. Disintegration and dissolution have been reported in other studies as important indicators of poor drug quality [47], [51], [52]. A more comprehensive quality assessment, as recommended by the US Pharmacopoeia [50] and implemented in Amazon countries [47], should be considered in subsequent investigations. Systematic visual inspection of the packaging was not included as some samples were repacked from large jars in health facilities. Inspection of the packages might have helped to classify the poor-quality samples. Classification as counterfeit (intellectual property infringement) or falsified (both considered deliberate actions), substandard (e.g. as a result of poor manufacturing practice) or degraded is important in order to identify appropriate solutions [17]. Responses from manufacturers confirmed the authenticity of certain poor-quality products but whether the low chemical content in the samples was a result of poor manufacturing, negligence, or deliberate action cannot be judged based on the available information. Poor-quality medicines were ubiquitous in the health facility supply chain and as commonly found in central warehouses and hospitals as in remote aid posts that often lack appropriate storage facilities. This suggests that poor-quality medicines identified in this study are unlikely to be a result of inadequate transport and storage conditions, but rather of poor manufacturing.

In the absence of any systematic sampling of medicines for quality control [27] and only two previous small scale studies [29], [37], this survey including samples from across the country is the most representative and comprehensive data on medicines quality in PNG available to date. The inclusion of providers at all levels of the supply chain adds extra value to the data and the overt collection of medicines is unlikely to have introduced a major bias under the assumption that the health facility officers-in-charge were unaware of the quality of individual medicines on their shelves. A collection of additional essential medicines could have provided a more comprehensive picture of medicine quality in PNG health facilities, but was beyond the scope of this study. Nationwide surveys on medicine quality often focus on private sector providers which are generally less well regulated and have been found to sell poor-quality drugs [17], [30], [53]. The importance of including the health facility sector in such studies is reflected in our finding of poor-quality products, even though extending the sampling of medicines to private sector providers across PNG would be an important next step.

The wide-spread availability of poor-quality medicines in formal health facilities reflects the lack of adequate quality control and regulatory mechanisms in PNG [27]. As a consequence, any type of poor-quality medicine, including fake artemisinin derivatives that have in the past been found in southeast Asia [49], [53], could in the future easily enter and spread through the local medicines market. The fact that only two sampled medicines (both artemether-lumefantrine) were WHO prequalified products calls into question the standards and procedures applied in the central procurement of medicines. Poor-quality samples of primaquine from one manufacturer (BDH Industries) were label “NVBDCP [Indian National Vector Borne Disease Control Program] SUPPLY, NOT FOR SALE” raising further questions about drug supply channels (Figure S1).

In order to protect patients and safeguard the efficacy of medicines, concerted global and local efforts are necessary [11], [54]. Clinical practitioners and pharmacists in PNG should be aware that the availability and inadvertent administration of poor-quality medicines could result in an increase in morbidity or mortality due to malaria or sepsis. Given the difficulties in collecting valid relevant aggregate data on drug quality and the likely delay in analysing the results and promulgating the findings, the present data suggest that individual PNG clinicians should include poor-quality medicine as part of the differential diagnosis of repeated unexpected treatment failure with a particular therapy, not only in the case of malaria. We recommend that alternative formulations are sourced and used in this situation, that samples of the suspect treatment are retained for subsequent content analysis in a central facility, and that these measures are communicated to the National Department of Health so that wider action can be considered. At the same time, urgent measures are required to stop the availability of poor-quality products in both the public and the private sector. First steps should include limiting procurement of drugs to WHO prequalified products and implementing routine quality testing. Basic low-tech test procedures can be used as a cost-effective means to routinely screen medicines arriving in the country and to test samples collected from peripheral facilities and retailers, without requiring advanced technology [50], [55]. Sophisticated analyses based for example on HPLC, while unlikely to be cost-effective and sustainable for high numbers of samples [10], should be applied to validate the basic test results and provide more in-depth information on poor-quality medicines [47], [50].

## Supporting Information

Figure S1 Photographs of packages containing poor-quality medicines.
(PDF)

Table S1 API content results for primaquine samples measured by HPLC and LC-MS.
(XLSX)

Table S2 Details of all samples containing less or more than the acceptable amount of API.
(XLSX)
