# Supplementary material for: Quality of Antimalarial Drugs and Antibiotics in Papua New Guinea: A Survey of the Health Facility Supply Chain
Source: PLoS One. 2014 May 14;9(5):e96810. doi: 10.1371/journal.pone.0096810 (PMC4020934; doi:10.1371/journal.pone.0096810)
Supplement: Figure S1 — Photographs of packages containing poor-quality medicines. (PDF) [file pone.0096810.s001.pdf]

Figure S1. Photographs of packages containing poor-quality medicines.

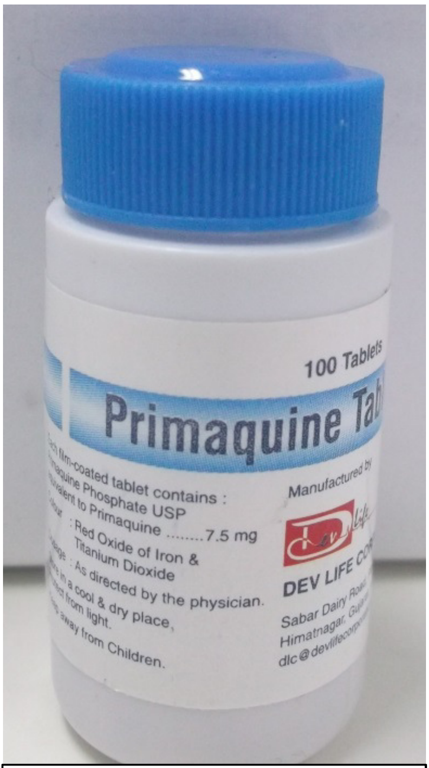

Primaquine, ID 15
Dev Life Corporation

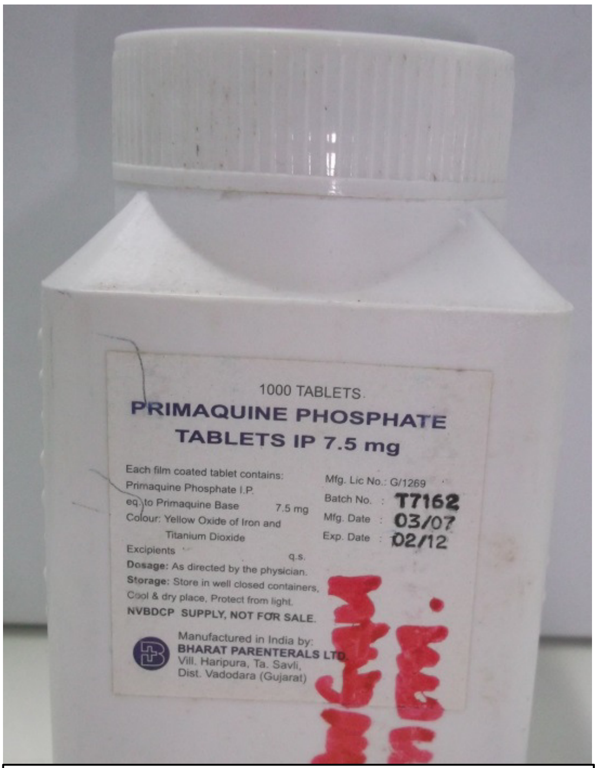

Primaquine, ID 346
Bharat Parenterals

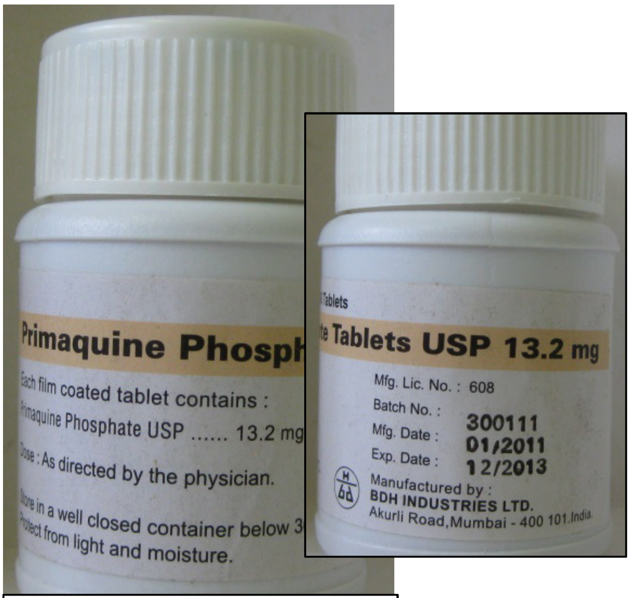

Primaquine, ID 124
BDH Industries

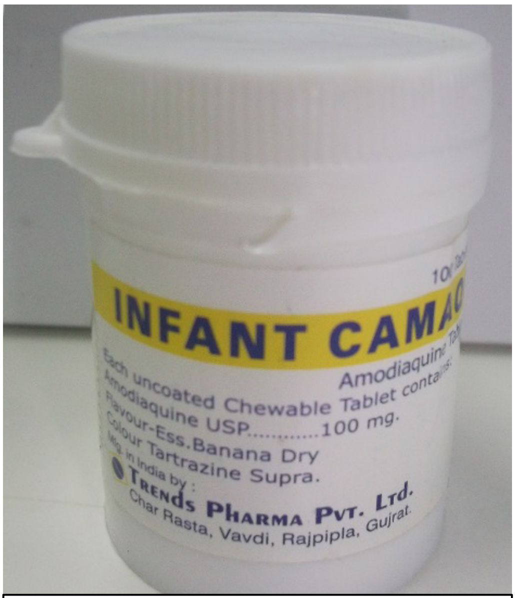

Amodiaquine, ID 24
Trends Pharma
